# Longitudinal Genome‐Wide Association Study for Female Fertility Traits in German Holstein Cattle

**DOI:** 10.1002/age.70078

**Published:** 2026-02-11

**Authors:** S. Sakhaeifar, T. Yin, S. König

**Affiliations:** ^1^ Institute of Animal Breeding and Genetics Justus‐Liebig‐University of Gießen Gießen Germany; ^2^ Zhejiang Key Laboratory of Dairy Cattle Genetic Improvement and Milk Quality Research Wenzhou P.R. China

**Keywords:** female fertility traits, gene identifications, longitudinal GWAS

## Abstract

The aim of this genome‐wide association study (GWAS) was to detect significant SNP effects influencing the dynamic process of female fertility in dairy cattle over lactations, to identify possible candidate genes and to study their role in pathway analyses. We considered records for the female fertility traits non‐return rate after 56 days (NRR56), interval from calving to first service (CTFS) and days open (DO) up to parity six from 190 269 lactating Holstein cows and heifers. The longitudinal GWAS followed a 2‐step approach. In step 1, we estimated (co)variance components by combining pedigree and genomic relationships in random regression models with additive‐genetic and permanent environmental regressions on the time‐dependent gradient ‘parity’. The matrix for estimated (co)variance components for random regression coefficients from step 1 was integrated into the longitudinal GWAS in step 2 to estimate SNP effects and significance for (a) the outer ‘layer’ representing baseline effects (intercept), (b) the middle ‘layer’ representing the slope and (c) the inner ‘layer’ indicating significant SNPs in all lactations, but with differing effects. For the ‘inner layer’, we detected the following five potential candidate genes: *TMEM132C* and *IMPG1* for NRR56, *DCHS2* for CTFS and *CSMD1* and *CSNK1A1* for DO. The identified genes also play a dominant role in biological pathways related to physiological fertility mechanisms. Overall, the longitudinal GWAS illustrated the dynamic genetic mechanisms of gene regulations on female fertility traits with progressing time.

## 1 Introduction

Fertility plays an essential role in dairy cow farming systems, with direct impact on the herd reproduction status, indirect effects on production traits and ultimately determining overall profitability (Shalloo et al. 2014). From a breeding perspective, improvements in female fertility traits strongly determine intra‐herd replacement rates, with causal effects on selection intensities (VanRaden et al. 2004). In the context of the interplay between production and reproduction, high reproduction rates ensure the onset of new lactations with high milk production after calving (Beerda et al. 2008; Pinedo and de Vries 2010; Kiser et al. 2019). However, genetic antagonistic relationships among production and female fertility traits have been reported in numerous studies (e.g., Gernand and König 2017).

Female fertility traits are typically polygenic, implying that they are controlled by multiple genes, each contributing small individual effects (Gajbhiye et al. 2018). The expression of these fertility‐related genes can vary throughout an animal's lifespan, with some genes switching on or off during different life stages, making them more challenging to detect (McGrath et al. 2021). Environmental factors related to, for example, nutrition, management practices and herd hygiene status also play substantial roles in shaping fertility outcomes, partly with genetic interactions. Strong environmental influences can overshadow genetic contributions, complicating the use of genetic effects in selective breeding programmes (Sakali et al. 2024).

In recent years in the genomic era, based on the availability of dense high‐throughput SNP marker data, genome‐wide association studies (GWAS) have been widely applied to identify genetic variants for female fertility traits including the fertilisation success at first service (Galliou et al. 2020), pregnancy rates (Wright et al. 2020) and non‐return rates (Frischknecht et al. 2018). The detection of a broad pattern of potential candidate genes associated with the above‐mentioned female fertility traits in Holstein‐Friesian cows supported the infinitesimal model of inheritance.

Inferring genetic mechanisms remains a challenge, because female fertility is a dynamic trait with alterations and fluctuations across life stages and reproductive cycles. The genetic mechanisms influencing fertility may shift over time, with different genes being expressed at varying stages of lactation, as well as across lactations. As outlined by Das et al. (2011), traditional GWAS to capture static genetic effects do not fully account for such dynamic variation. To address this limitation in a quantitative‐genetic context, Schaeffer (2004) introduced the applications of random regression models (RRM) for a more accurate estimation of genetic parameters across lactation stages, age classes and parities. As a further extension based on dense genomic marker data, so‐called ‘longitudinal GWAS’ were suggested to infer SNPs with altering effects in progressing time, for example, to capture the dynamic processes of disease processes in humans (Wiegrebe et al. 2024). Applying this approach in an animal genetics context, Ning et al. (2018) studied SNP effects at multiple time points, providing a comprehensive understanding of altering genetic mechanisms for milk production of Chinese Holstein cows. Unlike traditional GWAS, which assume constant effects for each SNP, longitudinal GWAS capture the dynamic nature of genetic influences over time, for example, across lactations. In longitudinal GWAS, only a limited number of SNPs are expected to exhibit significant effects over the whole time trajectory, implying the crucial importance of the SNP × time interaction term (Sikorska et al. 2015). Sikorska et al. (2015) applied linear mixed models to longitudinal phenotypes to estimate effects for the slope, and in a subsequent step, slope estimates were considered as dependent variables in genomic analyses. Wendel et al. (2021) conducted longitudinal GWAS in a single step by defining linear mixed models with a SNP × time interaction term. Ning et al. (2018) initially estimated the variance components from a random regression model, and subsequently, these estimates were considered as phenotypes in the model for whole‐genome association analyses. Such approaches are especially relevant for female fertility traits, where gene expression and regulatory mechanisms are known to fluctuate throughout the dairy cow's reproductive cycle (Pimentel et al. 2011), suggesting the importance of different genes in different lactations.

Consequently, the aim of this study was to infer SNP effects for female fertility traits in German Holstein cows considering the physiological dynamics from an across‐lactation perspective by applying a longitudinal genome‐wide association approach and considering the (co)variance components for additive‐genetic and permanent environmental effects from a genomic random regression model. Subsequently, significant SNPs for the longitudinal component influencing female fertility across lactations were used to identify candidate genes and explore the functional and biological roles of these genes and their relevance to regulatory processes of female fertility.

## 2 Materials and Methods

### 2.1 Data

#### 2.1.1 Female Fertility Traits

The phenotypic dataset for the female fertility traits included records for the non‐return rate after 56 days (NRR56), for the interval from calving to first service (CTFS), and for days open (DO) from 190 269 lactating cows and heifers kept in 45 large‐scale contract herds located in the German federal states of Hesse, Berlin‐Brandenburg and Mecklenburg‐West Pomerania. Female fertility traits were recorded in the calving years 2011–2022. The number of cattle by parity and trait is given in Table 1. Phenotypic averages were 0.71 (SD = 0.45) for NRR56 in heifers, 0.49 for NRR56 in cows (SD = 0.49), 78.24 days for CTFS (SD = 27.50) and 122.08 days for DO (SD = 60.91). Descriptive female fertility trait statistics by parity indicating the phenotypic trend across ages are shown in Table 1.

**TABLE 1:** Number of observations and descriptive statistics for the female fertility traits non‐return rate after 56 days (NRR56), interval from calving to first service (CTFS) and days open (DO) by parity (parity 0 = heifers).

Trait	Parity	No. of observations	Mean	SD
NRR56	0	166 736	0.71	0.45
CTFS	0	0	—	—
DO	0	0	—	—
NRR56	1	154 352	0.53	0.49
CTFS	1	158 132	77.09	27.40
DO	1	128 808	117.43	58.75
NRR56	2	114 446	0.49	0.50
CTFS	2	117 442	77.57	26.86
DO	2	89 513	123.00	60.15
NRR56	3	146 790	0.47	0.49
CTFS	3	150 519	79.97	27.15
DO	3	102 042	127.13	60.21
NRR56	4	41 938	0.46	0.49
CTFS	4	43 076	80.45	27.33
DO	4	28 808	128.67	60.73
NRR56	5	20 958	0.47	0.49
CTFS	5	21 512	81.23	27.28
DO	5	13 346	128.94	59.76
NRR56	6	9127	0.46	0.49
CTFS	6	9380	81.22	26.93
DO	6	5503	128.76	59.37

#### 2.1.2 Genotypes and Pedigree

**FIGURE 1:**
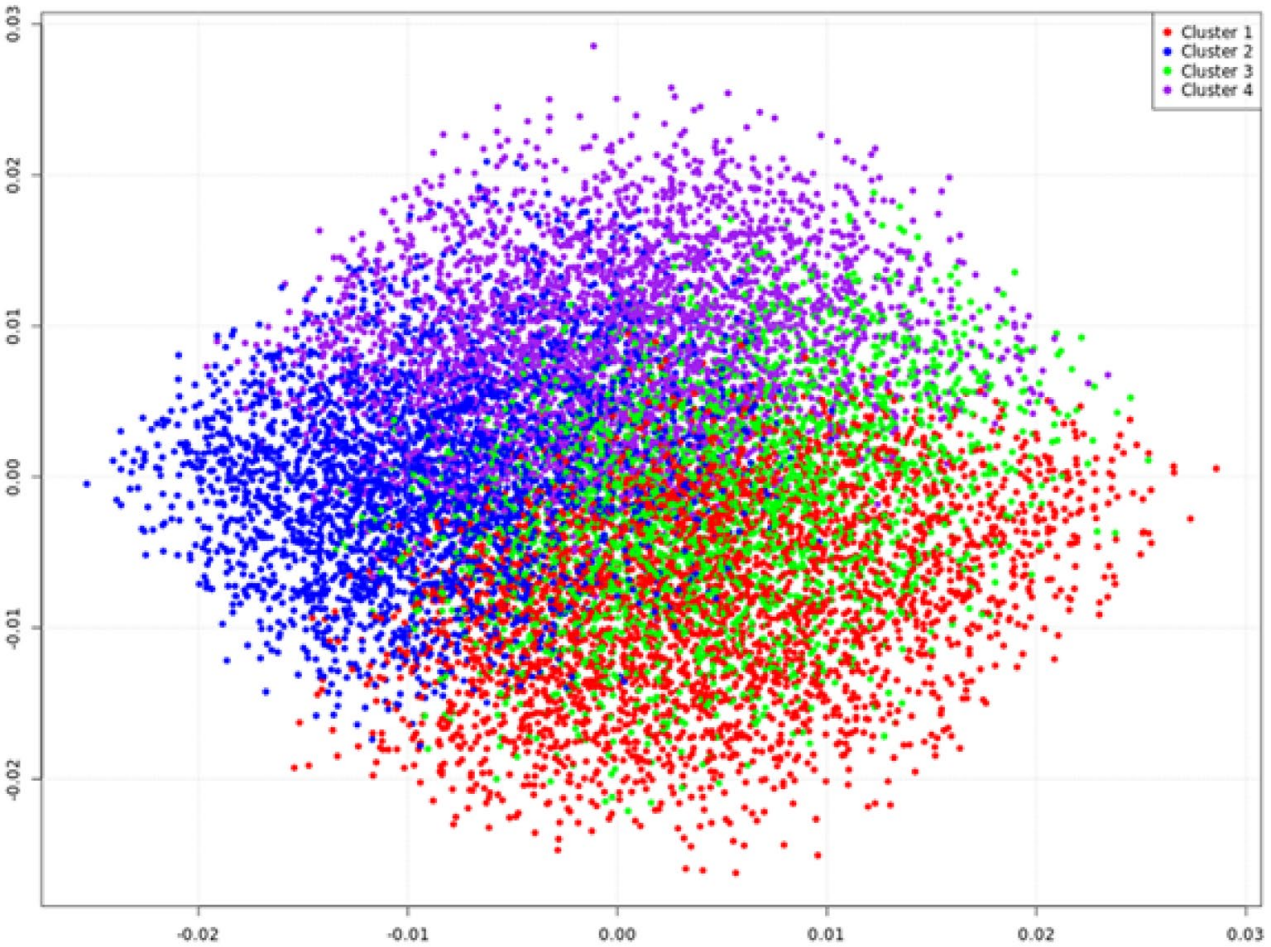
Plot of the first two principal components (PC 1 and PC 2) of the genomic relationship matrix for the genotyped female cattle. The four different clusters represent the allocation to four groups based on *k*‐means clustering considering the first five PCs of the genomic relationship matrix.

The genotype dataset included 21 316 cows with phenotypes for all three female fertility traits. In this regard, 5403 cows were genotyped using the *Illumina Bovine SNP50 v2 BeadChip* and 15 913 cows were genotyped using the *Illumina Bovine Eurogenomics 10 K* low‐density chip. The genotypes obtained from the 10 K chip were subsequently imputed to the 50 K panel in the routine process of national genetic evaluations for German Holstein (Segelke et al. 2012). Quality control (QC) of the genotype data was performed using the preGSf90 programme, as implemented in the BLUPf90 software package (Misztal et al. 2018). The filters applied included a minimum minor allele frequency (MAF) threshold of 0.05, a minimum call rate of 0.9, detection of deviation from Hardy–Weinberg equilibrium (*p*‐value < 10^−6^), exclusion of animals with genomic relationships exceeding 0.95, resolution of Mendelian conflicts (i.e., inconsistencies between genotypes of parents and offspring), and elimination of monomorphic SNPs. After QC, the dataset consisted of 41 129 SNPs from 21 048 genotyped cows. The genomic relationship matrix for these animals was constructed applying the algorithm of VanRaden et al. (2004). The principal components (PCs) from the genomic relationship matrix considering the female cattle with phenotypes were generated by applying the software package GCTA (Yang et al. 2011). PC1 and PC2 explained 11.6% and 9.7% of the total genetic variation, respectively. Afterwards, *k*‐means clustering was applied by considering the first five PCs to allocate the animals to four groups with similar genomic relationships. The plot for the first two PCs (Figure 1) and the smooth gradient observed between clusters confirm that the genotyped cows belong to a largely homogeneous population, implying that we did not need to account for further population stratification in the genome‐wide association analysis.

The pedigree of the phenotyped 190 269 female cattle comprised at least three generations backwards on the maternal as well as on the paternal side, with 5787 different sires, 7116 different maternal grand‐sires, and 2850 different paternal grand‐sires. Oldest founder animals were from the birth year 1920.

### 2.2 Statistical Models

We applied a two‐step strategy. In the first step, we combined genomic and pedigree data to estimate (co)variance components via RRM. The estimated (co)variance components for random regression coefficients and further random effects were used as input data in the longitudinal GWAS in step 2.

#### 2.2.1 Estimation of Variance Components

The single‐trait RRM (model 1, consecutive runs for the three traits NRR56, DO and CTFS) in matrix notation for the estimation of (co)variance components was the following:

(1) y=Xb+Qu+Zp+Ss+e

where **y** = vector for the observations for NRR56, DO or CTFS in different lactations, **b** = vector for fixed effects including herd‐year‐season of insemination, insemination age in months and linear regressions on scaled lactation number (first order Legendre polynomials with intercept and slope); **u** = vector of additive genetic effects for random regression coefficients on the scaled lactation number (first order Legendre polynomials with intercept and slope) **u** ~ *N* (0, **H** ⊗ **G**), and **H** denoting the combined (pedigree and genomics) relationship matrix constructed according to Legarra et al. (2009), ⊗ representing the Kronecker product and **G** = (co)variance matrix for random regression coefficients of additive genetic effect; **p** = vector of permanent environmental effects for random regression coefficients on the scaled lactation number (first order Legendre polynomials with intercept and slope) with **p** ~ *N* (0, **I** ⊗ **P**) and **P** = (co)variance matrix for random regression coefficients of permanent environmental effects, **s** = vector for the random service sire effects (for NRR56 and DO) with **s** ~ *N* (0, **I** $\sigma_{s}^{2}$) and $\sigma_{s}^{2}$ denoting the service sire variance; and **e** = vector for the random residual effects with **e** ~ *N* (0, **I** $\sigma_{e}^{2}$) and $\sigma_{e}^{2}$ denoting the residual variance; and **X**, **Q**, **Z** and **S** = the respective incidence matrices. The concept of random regression modelling with intercept and slope is based on the reaction norm approach to study environmental sensitivity in German Holstein cows by Streit et al. (2012), but adapted to a temporal perspective in the present study.

#### 2.2.2 Longitudinal Genome‐Wide‐Associations

Step 2 was the longitudinal GWAS, which was restricted to phenotyped female cattle with genotypes, due to the challenges of the algorithms presented below and computation time limitations. The respective datasets for the longitudinal GWAS included 38 939 records for NRR56, 38 398 records for CTFS and 22 740 records for DO. The model 2 for the longitudinal GWAS in matrix notation was

(2) $$y=\mathrm{Xb}+X_{\mathrm{snp}}b_{\mathrm{snp}}+\mathrm{Qu}+\mathrm{Zp}+\mathrm{Ss}+e$$

where **y** = the vector of observations for NRR5, CTFS and DO; **b**_**snp**_ = vector for fixed effects for 41 129 SNPs and **X**_**snp**_ = the related incidence matrix for SNPs considering the three possible SNP‐genotypes. The remaining effects were the same as defined for model 1.

For solving the equations of model 2 with regard to SNP effects and significance levels, we followed the ‘Eigen decomposition’ approach according to Ning et al. (2018), by applying our own R code (R Core Team 2023) (Appendix S1). The technical description of the respective workflow is outlined in Appendix S2.

### 2.3 Candidate Gene Identification

Candidate genes were identified based on the significant SNP effects from the inner layer of the circular Manhattan plots. We applied the biomaRt R package from Bioconductor to retrieve the rs accession numbers of SNPs associated with the traits of interest, using the getBM() function (Durinck et al. 2005, 2009). Candidate genes were identified and mapped to these SNPs based on the latest gene annotations from ENSEMBL (McLaren et al. 2016), using the *Bos taurus* ARS‐UCD1.2 genome assembly (Yates et al. 2020). A gene was considered as a candidate if at least one SNP exceeding the *Psug* threshold was located either within the gene or within a 200 kb window (100 kb upstream and 100 kb downstream). The identified potential candidate genes were manually submitted to DAVID version 2021 (Huang et al. 2009; Sherman et al. 2022) to retrieve Gene Ontology (GO) terms and associated biological functions. In addition, the Bgee database (Bastian et al. 2021) was queried to obtain gene expression scores, that is, values for these genes quantifying their activity in a specific cattle tissue sample.

### 2.4 Pathway Analysis Including the Identified Genes

For the investigations of gene expressions and associated biological pathways, we utilised the SRplot online platform for pathway analysis, visualisation and graphing (Tang et al. 2023), which uses the Kyoto Encyclopedia of Genes and Genomes (KEGG) database (Kanehisa and Goto 2000). This approach enabled us to identify KEGG pathways related to the identified genes in *B. taurus*.

## 3 Results

### 3.1 Variance Components

The estimates for (co)variance components from the RRM (model 1) are displayed in Table 2. The (co)variance components comprise the intercept and slope for additive‐genetic effects and for permanent environmental effects, and the variances for the service sire and residual components. The matrices for these (co)variance components were input data for the algorithm of the subsequent longitudinal GWAS in step 2. Considering the variance components, the heritability for NNR56 was 0.010 in parity 0 for heifers, 0.011 in parity 1, 0.013 in parity 2, 0.017 in parity 3, 0.023 in parity 4, 0.030 in parity 5 and 0.038 in parity 6. The SE of the NRR56 heritability estimates ranged from 0.001 (heifers) to 0.003 (cows from parity 6). For CTFS, the heritability was 0.072 in parity 1, 0.053 in parity 2, 0.047 in parity 3, 0.052 in parity 4, 0.066 in parity 5 and 0.091 in parity 6, with SE in the range from 0.015 (parity 1) to 0.022 (parity 6). The heritability for DO was 0.037 in parity 1, 0.026 in parity 2, 0.022 in parity 3, 0.021 in parity 4, 0.024 in parity 5 and 0.031 in parity 6. The SE for the DO heritabilities ranged from 0.013 (parity 1) to 0.020 (parity 6).

**TABLE 2:** Variance and covariance components for the traits non‐return rate after 56 days (NRR56), calving to first service (CTFS) and days open (DO).

Trait	(Co)variance components							
	var_a1_	cov_a_	var_a2_	var_p1_	cov_p_	var_p2_	var_s_	var_e_
NRR56	0.0033	0.0013	0.0006	0.0048	0.004	0.0034	0.0023	0.2235
CTFS	16.43	2.56	8.15	58.19	29.41	19.09		411.87
DO	128.04	69.83	63.69	67.88	92.91	138.67	19.41	2576.88

Abbreviations: cov_a_, additive genetic covariance between the intercept and slope; cov_p_, phenotypic covariance between the intercept and slope; var_a1_, additive genetic variance for the intercept (baseline genetic variance); var_a2_, additive genetic variance for the slope (change in genetic variance over time); var_e_, residual variance; var_p1_, phenotypic variance for the intercept (baseline phenotypic variance); var_p2_, phenotypic variance for the slope (change in phenotypic variance over time); var_s_, service sire variance.

The genetic correlations between the same traits from different parities from the RRM (model 1) were high for adjacent cow parities, but gradually declined with increasing parity distance. The genetic correlations between NRR56 in parity 1 with NRR56 in parities 2, 3, 4, 5 and 6 were 0.89, 0.79, 0.70, 0.69 and 0.67, respectively. Smaller genetic correlations in the range from 0.54 to 0.65 were identified between NRR56 in heifers with NRR56 in all cow parities. Genetic correlations between CTFS from adjacent parities exceeded 0.91, and those between DO from adjacent parities exceeded 0.90. As for NRR56, the genetic correlations for CTFS and DO declined with increasing parity distances.

### 3.2 Longitudinal Genome‐Wide Associations

**FIGURE 2:**
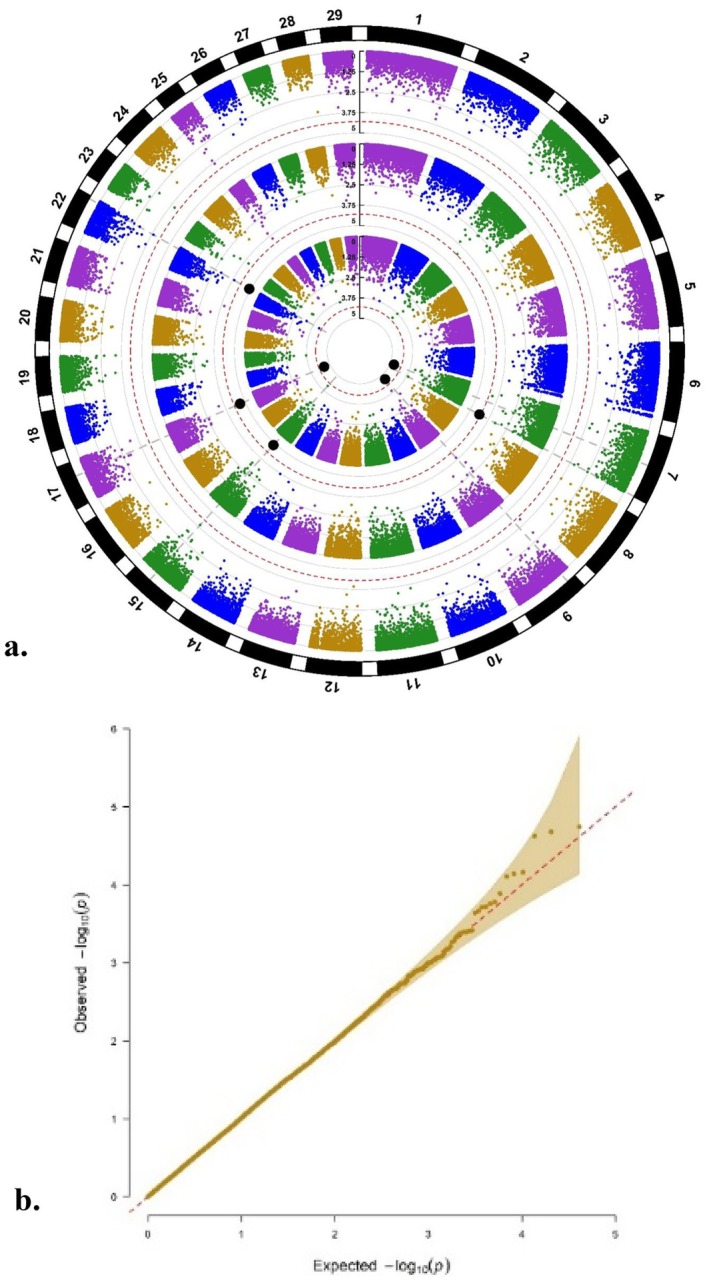
(a) Circular Manhattan plot for the non‐return rate after 56 days (NRR56) displaying the *p*‐values of SNPs for the three ‘layers’ intercept (outer layer), slope (middle layer) and the combination of both (inner layer) (the dotted red circular line for each layer represents the respective suggestive threshold and the enlarged black dots the significant SNPs). (b) *Q*–*Q* plot illustrating observed *p*‐values plotted versus expected *p*‐values, highlighting any deviation from the null hypothesis (*λ* = 1.01).

**FIGURE 3:**
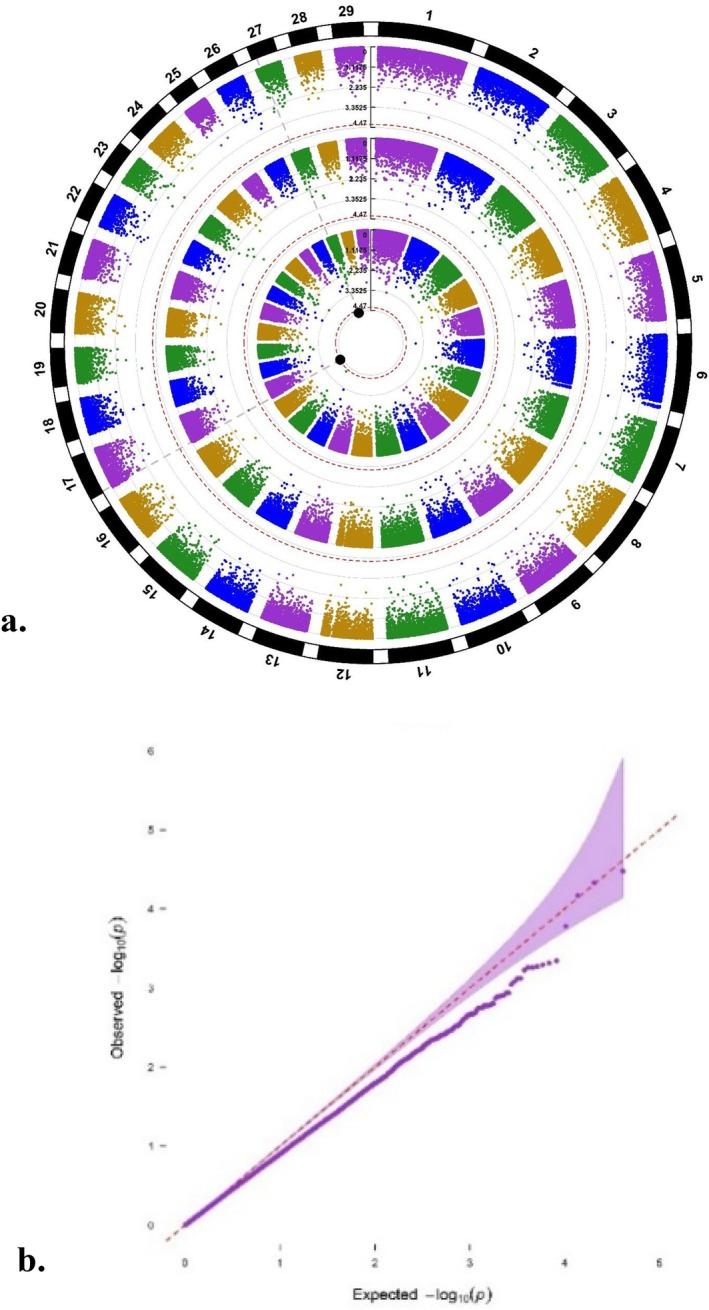
(a) Circular Manhattan plot for the interval from calving to first insemination (CTFS) displaying the *p*‐values of SNPs for the three ‘layers’ intercept (outer layer), slope (middle layer), and the combination of both (inner layer) (the dotted red circular line for each layer represents the respective suggestive threshold and the enlarged black dots the significant SNPs). (b) *Q*–*Q* plot illustrating observed *p*‐values plotted versus expected *p*‐values, highlighting any deviation from the null hypothesis (*λ* = 0.91).

**FIGURE 4:**
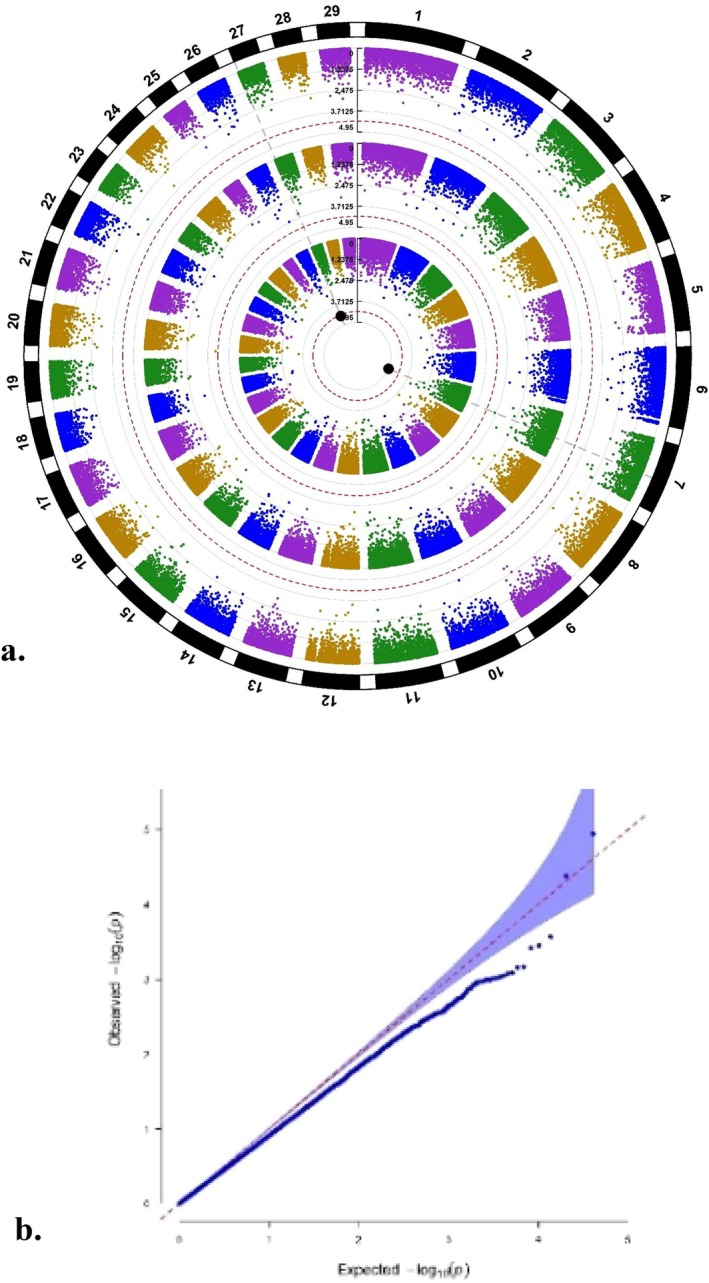
(a) Circular Manhattan plot for days open (DO) displaying the *p*‐values of SNPs for the three ‘layers’ intercept (outer layer), slope (middle layer), and the combination of both (inner layer) (the dotted red circular line for each layer represents the respective suggestive threshold and the enlarged black dots the significant SNPs). (b) *Q*–*Q* plot illustrating observed *p*‐values plotted versus expected *p*‐values, highlighting any deviation from the null hypothesis (*λ* = 0.9).

The circular Manhattan plots for the SNP effects and the respective *Q*–*Q* plots from the longitudinal GWAS are displayed in Figure 2 for NRR56, in Figure 3 for CTFS and in Figure 4 for DO. With regard to NRR56 and the inner layer for the longitudinal GWAS, no SNP variant surpassed the 𝑃*bonf* threshold. However, three SNPs including ARS‐BFGL‐NGS‐116933 (rs109487947) at BTA 7:55,181,283, BTB‐00380633 at BTA 9:15,966,478 and ARS‐BFGL‐NGS‐104247 (rs109066308) at BTA 17:49,578,439 were associated according to *𝑃sug*. The genomic inflation factor (𝜆) of 1.01 and the *Q*–*Q* plot (included in Figure 2) indicated unbiased estimates. The three significant SNPs for the inner layer explained 3.56% (rs109487947), 0.23% (BTB‐00380633) and 0.11% (ARS‐BFGL‐NGS‐104247) of the total genetic variation for NRR56. No single SNP was significant for the outer layer (intercept). Associated SNPs according to *𝑃sug* for the middle layer (slope) included the variants ARS‐BFGL‐NGS‐84234 (rs109703134) at BTA 7:111,655,507, ARS‐BFGL‐NGS‐59605 (rs109476806) at BTA 15:52,762,755, ARS‐BFGL‐NGS‐104247 (rs109066308) at BTA 17:49,578,439 and ARS‐BFGL‐NGS‐62254 (rs42782475) at BTA 22:55,770,123. Notably, ARS‐BFGL‐NGS‐104247 (rs109066308) on BTA 17 was significant for both the middle and inner layer. The significant SNPs for the middle layer explained between 0.10% and 4.03% of the total genetic variation for NRR56. The correlation coefficient between SNP effects for the intercept and slope for NRR56 was 0.87, indicating stability of associated SNPs across all lactations while displaying changing effects over time.

With regard to CTFS, no SNP surpassed the significance threshold for the outer layer (intercept) or the middle layer (slope) (Figure 3). According to *𝑃sug*, the two significant SNPs for the inner layer were BTA‐109611 (rs41572882) at BTA 17:3,009,412 and Hapmap51513‐BTA‐88215 (rs41596684) at BTA 27:8,689,042. The genomic inflation factor (𝜆) was 0.91, indicating slight but acceptable deflation, as displayed in the corresponding *Q*–*Q* plot (Figure 3). The two significant SNPs for the inner layer explained 0.10% (rs41572882) and 0.08% (rs41596684) of the total genetic variation for CTFS. The correlation coefficient between SNP effects for the intercept and for the slope was 0.72.

The Manhattan plots for the three layers for DO are shown in Figure 4. No single SNP surpassed the suggestive significance threshold for the outer layer (intercept) or the middle layer (slope). For the inner layer combining both effects (intercept and slope), no SNP exceeded the *𝑃bonf* threshold, but two SNPs surpassed the *𝑃sug* threshold, including UA‐IFASA‐7691 (rs41655307) at BTA 7:62,860,816 and ARS‐BFGL‐NGS‐1430 (rs109833308) at BTA 27:1,467,500. The genomic inflation factor (𝜆) was 0.90, indicating slight but acceptable deflation. The *Q*–*Q* plot confirmed the absence of inflation (Figure 4). The two significant SNPs for the inner layer explained 1.13% (rs41655307) and 0.15% (rs109833308) of the total genetic variation for DO. The correlation coefficient between SNP effects for intercept and slope was 0.83.

### 3.3 Identification of Potential Candidate Genes and Their Roles in Biological Pathways

Genes located within 100 kb windows of significant or suggestive SNP positions were considered as candidate genes. For example, the SNP ARS‐BFGL‐NGS‐116933 (rs109487947) was excluded due to its location in an intergenic region, outside any known gene boundaries or regulatory elements.

The identified potential candidate genes for the three female fertility traits along with the respective locations of the identified significant SNPs and brief descriptions of the gene functions are listed in Table 3. The GO terms and biological functions of these five genes as retrieved from DAVID, and the respective gene expressions scores are shown in Table 4. With regard to NRR56, one significant variant, ARS‐BFGL‐NGS‐104247 (rs109066308) on BTA 17, is located within the *TMEM132C* gene, which encodes transmembrane protein 132C. *TMEM132C* serves as a neural adhesion molecule due to its structural features, including cohesion and immunoglobulin domains. These domains are characteristic of proteins involved in cell adhesion processes, which are vital for neural development and function. While specific pathways involving *TMEM132C* have not been fully elucidated in cattle, its potential role in cell adhesion with GO:0016020 ~ membrane and the expression scores of 72.20 in the fornix of vagina, 71.90 in the uterine cervix and 62.47 in the isthmus of fallopian tube suggest influence in reproductive processes by affecting cell–cell interactions within the reproductive tract. Another potential candidate gene for NRR56 is *IMPG1* on BTA 9, within the window of SNP BTB‐00380633. *IMPG1* is well‐known for its role in the visual system, contributing to the structural integrity of the interphotoreceptor matrix (GO:0007601 ~ visual perception, GO:0030198 ~ extracellular matrix organisation). The expression scores are 97.35 in the retina, 57.74 in the anterior segment of eyeball and 47.38 for the abomasum (Table 4).

**TABLE 3:** Identified potential candidate genes for non‐return rate after 56 days (NRR56), interval from calving to first service (CTFS) and days open (DO) based on the significant SNPs from the longitudinal GWAS.

Trait	SNP	rs‐id	Position	BTA	Identified gene	SNP loca‐tion^a^	Description
NRR56	ARS‐BFGL‐NGS‐104247	rs109066308	48550804	17	*TMEM132C*	yes	Transmembrane protein 132C
NRR56	BTB‐00380633	—	15766008	9	*IMPG1*	no	Interphotoreceptor matrix proteoglycan 1
CTFS	BTA‐109611	rs41572882	3027194	17	*DCHS2*	yes	Dachsous cadherin‐related 2
DO	ARS‐BFGL‐NGS‐1430	rs109833308	2599527	27	*CSMD1*	yes	CUB and Sushi multiple domains 1
DO	UA‐IFASA‐7691	rs41655307	60864104	7	*CSNK1A1*	yes	Casein kinase 1 alpha 1

^a^ Yes = SNP is located within the intron of the potential candidate gene; no = SNP is located in the 100 kb upstream or 100 kb downstream region of the potential candidate gene.

**TABLE 4:** Candidate genes associated with fertility traits, annotated with Gene Ontology (GO) biological processes using the DAVID tool (version 2021), and gene expression scores retrieved from the Bgee database.

Gene name	GO‐term and biological process	Gene expression score
*TMEM132C*	GO:0016020 ~ membrane, GO:0110165 ~ cellular anatomical entity	Fornix of vagina (72.20), uterine cervix (71.90), isthmus of fallopian tube (62.47)
*IMPG1*	GO:0007601 ~ visual perception, GO:0030198 ~ extracellular matrix organisation	Retina (97.35), anterior segment of eyeball (57.74), abomasum (47.38)
*DCHS2*	GO:0007156 ~ haemophilic cell adhesion via plasma membrane adhesion molecules, GO:0005509 ~ calcium ion binding, GO:0005911 ~ cell–cell junction, GO:0005886 ~ plasma membrane, GO:0072137 ~ condensed mesenchymal cell proliferation	Follicular cell of ovary (40.96), bone marrow (56.06), hypothalamus (47.49), theca cells (42.65) and granulosa cells (40.26)
*CSNK1A1*	GO:0006468 ~ protein phosphorylation, GO:0007030 ~ Golgi organisation, GO:0007049 ~ cell cycle, GO:0007165 ~ signal transduction, GO:0016055 ~ Wnt signalling pathway, GO:0016310 ~ phosphorylation, GO:0018105 ~ peptidyl‐serine phosphorylation, GO:0031670 ~ cellular response to nutrient, GO:0032436 ~ positive regulation of proteasomal ubiquitin‐dependent protein catabolic process, GO:0035025 ~ positive regulation of Rho protein signal transduction, GO:0045104 ~ intermediate filament cytoskeleton organisation, GO:0051301 ~ cell division, GO:0090090 ~ negative regulation of canonical Wnt signalling pathway, GO:1900226 ~ negative regulation of NLRP3 inflammasome complex assembly, GO:1904263 ~ positive regulation of TORC1 signalling	Neutrophils (98.23), oviduct epithelium (98.02) and milk (97.48)
*CSMD1*	GO:0001964 ~ startle response, GO:0007613 ~ memory, GO:0008584 ~ male gonad development, GO:0008585 ~ female gonad development, GO:0035846 ~ oviduct epithelium development, GO:0042593 ~ glucose homeostasis, GO:0060745 ~ mammary gland branching involved in pregnancy, GO:1990708 ~ conditioned place preference	Cumulus cells (61.43), oocytes (57.27), occipital lobe (53.07), prefrontal cortex (52.75), hypothalamus (52.57 in males, 52.35 in females), temporal cortex (47.45), pituitary gland (46.85) and cerebellum (46.67)

With regard to CTFS, the potential candidate gene *DCHS2* on BTA 17 harbours the significant SNP BTA‐109611 (rs41572882). *DCHS2* encodes the dachsous cadherin‐related protein 2. The expression scores of *DCHS2* in follicular cells of the ovary, in bone marrow, in the hypothalamus, in theca cells and in granulosa cells of female cows are 40.96, 56.06, 47.49, 42.65 and 40.26, respectively (Table 4). These follicular cells are key players in reproductive processes, including androgen secretion. GO‐analyses (Table 4) indicate that *DCHS2* is related to the following relevant terms: (a) nephron development (GO:0072006), suggesting its involvement in cellular differentiation and organ development, particularly in kidney formation, which may extend to reproductive health and (b) condensed mesenchymal cell proliferation (GO:0072137), which indicates the role of *DCHS2* in mesenchymal cell behaviour during organogenesis, potentially impacting reproductive tissues.

With regard to DO, the significantly associated SNP ARS‐BFGL‐NGS‐1430 (rs109833308) on BTA 27 is located within the *CSMD1* gene, which encodes CUB and Sushi multiple domains 1 (Table 3). *CSMD1* is expressed in several tissues and brain regions relevant to fertility and reproductive health in cows. As shown in Table 4, *CSMD1* shows moderate expression scores in cumulus cells (61.43) and oocytes (57.27), both with relevance for oocyte maturation and linked to GO:0035846 (oviduct epithelium development) and GO:0008585 (female gonad development), highlighting its potential role in oocyte quality and gonadal development. Additionally, *CSMD1* is moderately expressed in brain regions involved in stress and behavioural regulation of fertility, including the occipital lobe (expression score 53.07), prefrontal cortex (52.75), hypothalamus (52.57 in males, 52.35 in females), temporal cortex (47.45), pituitary gland (46.85) and cerebellum (46.67). These regions are associated with GO terms such as GO:0001964 (startle response), GO:0007613 (memory), GO:0008584 (male gonad development) and GO:0008585 (female gonad development), suggesting involvement in the hypothalamic–pituitary–gonadal axis and neuroendocrine pathways that influence reproductive function.

The second potential candidate gene for DO was *CSNK1A1* on BTA 7 harbouring the significant SNP UA‐IFASA‐7691 (rs41655307). The C*SNK1A1* gene encodes casein kinase 1 alpha 1 (Table 3). *CSNK1A1* is highly expressed in key reproductive tissues, such as neutrophils, oviduct epithelium and milk, with expression scores of 98.23, 98.02 and 97.48, respectively (Table 4). GO‐analysis indicated that *CSNK1A1* is involved in several biological processes, including protein phosphorylation (GO:0006468, GO:0016310), cell cycle regulation (GO:0007049) and signal transduction (GO:0007165). These processes are fundamental for proper cellular functions and are directly linked to reproductive health. *CSNK1A1* is also implicated in the regulation of the TORC1 signalling mechanisms (GO:1904263), which play a role in cell growth and metabolism and in the development of healthy oocytes.

**FIGURE 5:**
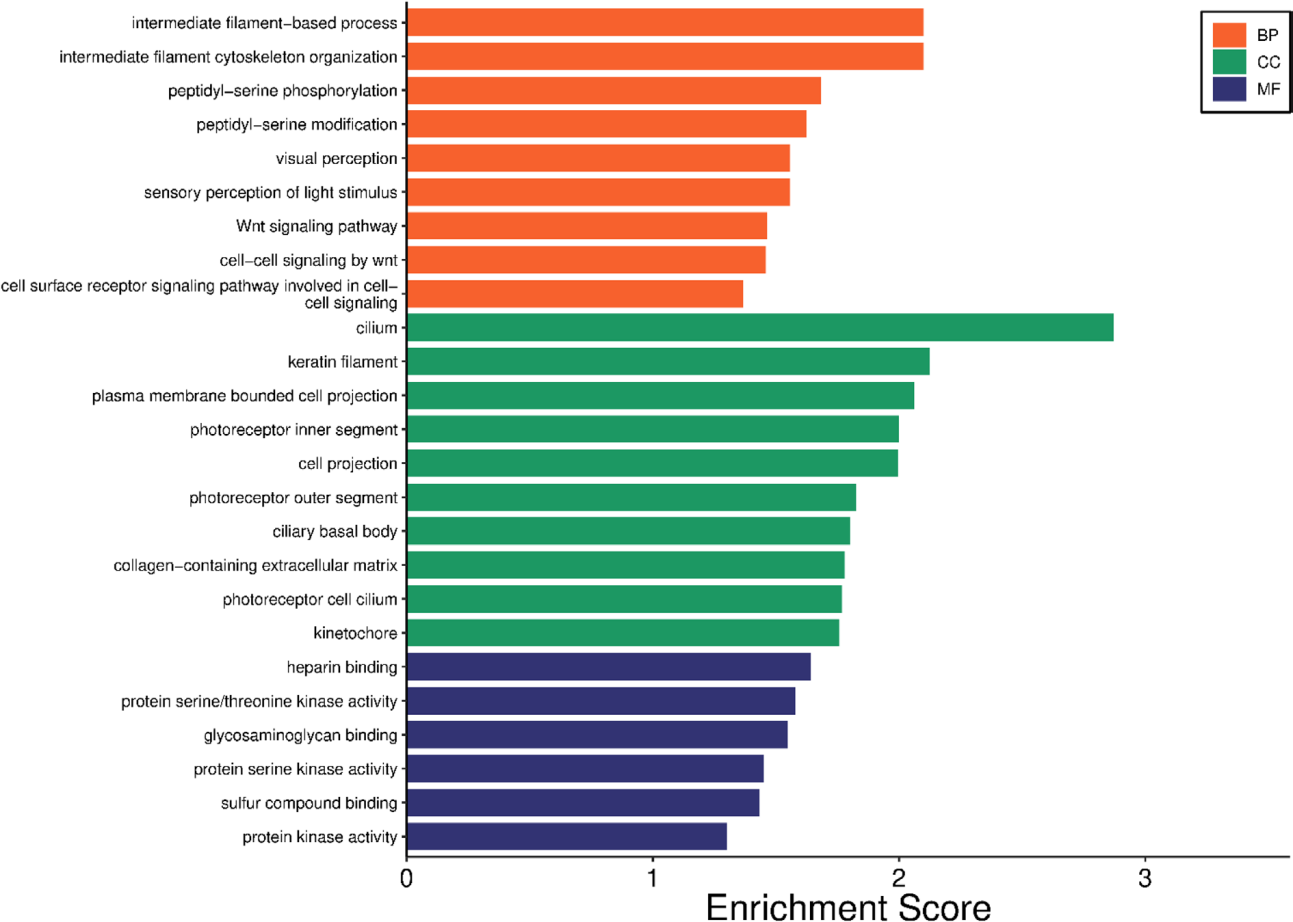
Gene Ontology (GO) enrichment analysis for fertility traits in dairy cows. The bar plot displays significantly enriched GO terms categorised into biological process (BP), cellular component (CC), and molecular function (MF).

The GO enrichment analysis revealed significant enrichment in all three GO domains: biological processes (BP), cellular components (CC) and molecular functions (MF), which are presented in Figure 5. Terms including the intermediate filament‐based process, intermediate filament cytoskeleton organisation and Wnt signalling pathway were enriched in the BP category, indicating a potential role in fertility processes. For the CC category, the terms cilium, keratin filament and photoreceptor segments were enriched, suggesting a role of cellular projections. For MF, enriched terms included heparin binding and protein kinase activity, highlighting potential regulatory and signalling functions. The genes *CSNK1A1* and *IMPG1* may play central roles in signal transduction and structural integrity related to reproductive processes. The convergence of structural and signalling pathways across GO categories supports the hypothesis that fertility‐associated genes function at multiple biological levels.

**FIGURE 6:**
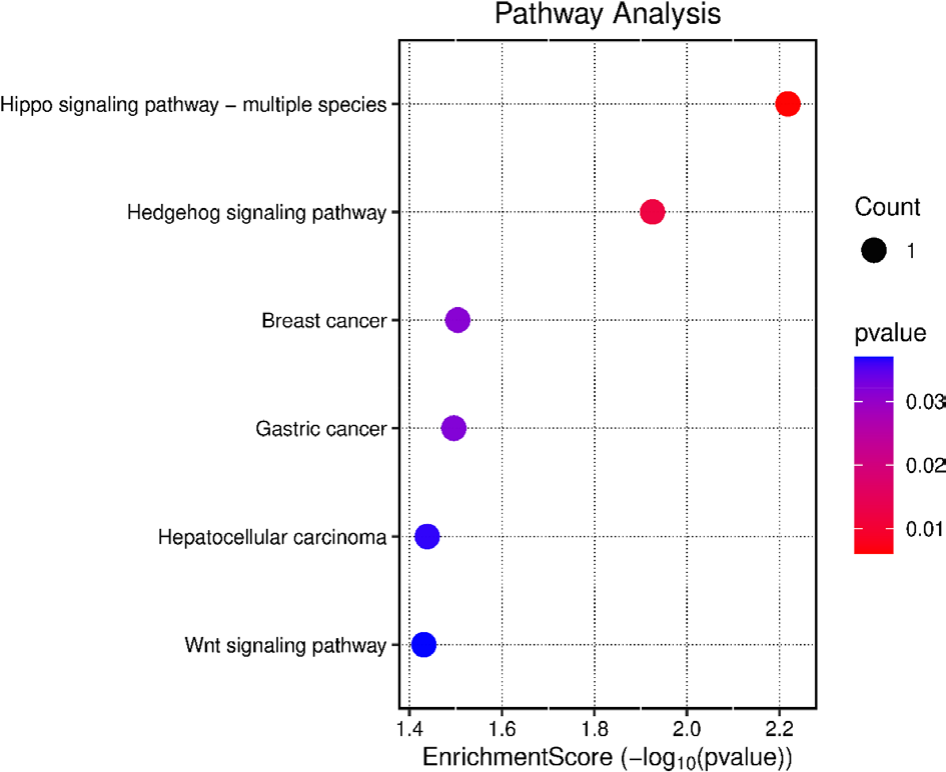
Pathway enrichment analysis of identified genes associated with the female fertility traits non‐return rate after 56 days, interval from calving to first insemination and days open. The plot indicates significantly enriched pathways, based on gene set analysis. The *y*‐axis represents pathways, the *x*‐axis shows the enrichment score (−log_10_ (*p*‐value)), and the dot size reflects the number of genes per pathway.

The pathway analysis, considering the identified candidate genes described above, suggests the involvement of several pathways related to regulation of key reproductive organs, female fertility and developmental functions, particularly highlighting the Hippo signalling pathway with an enrichment score of 2.2 (Table 5, with a visual illustration in Figure 6).

**TABLE 5:** Pathways related to specific KEGG (Kyoto Encyclopedia of Genes and Genomes) database entries based on identified candidate genes associated with female fertility traits days open (DO) and the interval from calving to first service (CTFS) and supported by previous studies from the literature.

Pathway	KEGG entry	Related trait	Identified candidate gene (BTA)	Same pathways addressed in other studies
Hedgehog signalling pathway	bta04340	DO	*CSNK1A1* (7)	Dilower et al. (2023)
Wnt signalling pathway	bta04310	DO	*CSNK1A1* (7)	Zhang et al. (2022), Hernandez Gifford (2015)
Hippo signalling pathway	bta04392	CTFS	*DCHS2* (17)	Clark et al. (2022), Lv et al. (2019), Plewes et al. (2019)
GnRH pathway	bta04912	DO	*CSNK1A1* (7)	Sun et al. (2022), Dai et al. (2022), Huo et al. (2022)

## 4 Discussion

### 4.1 Longitudinal Genome‐Wide Associations

The declining genetic correlations with increasing parity distances between the same traits from different parities suggest alterations of SNP effects for the same female fertility traits with progressing time. Specifically, the correlations between SNP effects for the outer layer reflecting the baseline effect (intercept) and the middle layer reflecting the slope were 0.87 for NRR56, 0.72 for CTFS and 0.83 for DO, indicating changes in the important SNPs with progressing time. However, the single SNP effects on the female fertility traits were generally small. Even the significant SNPs only explained at most 4.03% of the total genetic variation for the trait of interest (i.e., the effect of the SNP variant ARS‐BFGL‐NGS‐84234 on BTA 7 for NRR56).

In the past, many studies reported significant SNPs linked to fertility traits such as calving interval, days open and conception rate, providing valuable insights into the genetic basis of fertility (e.g., Höglund et al. 2015; Nayeri et al. 2016; Wolf et al. 2021). However, these studies assumed static effects of SNPs and potential candidate genes, overlooking dynamic genetic influences or temporal variations in gene expression across lactations. In our present study, the applied longitudinal GWAS identified seven SNPs associated with five potential candidate genes, influencing changes in fertility traits across parities. We explicitly focussed on the longitudinal genome‐wide associations (i.e., the inner layer of the circular Manhattan plot), which is a combination of intercept and slope, and considers the covariance between intercept and slope. In this regard, we identified three significantly associated SNPs for NRR56, two significantly associated SNPs for CTFS and two significantly associated SNPs for DO. A different set of significantly associated SNPs and important genomic segments for CTFS were reported by Wolf et al. (2021), who applied a ‘classical static’ approach for only one single record per cow. Mohammadi et al. (2020) focussed on same fertility traits, but they identified most of the significant SNPs on BTA 19, indicating the differences between ‘static’ and longitudinal GWAS for female fertility traits. However, other factors, such as differences between analysed populations, might explain the differences in genome‐wide associations for the same traits.

To the best of our knowledge, this is the first study focusing on longitudinal GWAS for female fertility traits in dairy cattle, allowing us to capture time‐dependent genetic effects and offering a more comprehensive understanding of fertility as a dynamic trait. Our approach for female fertility traits aligns with previous research by Sikorska et al. (2015) and by Ning et al. (2018), which emphasised the importance in incorporating time‐dependent genetic effects for a better and more precise understanding of complex traits. Ning et al. (2018) examined the genomic background of milk production traits and analysed a very dense longitudinal data structure based on daily measurements. In contrast, our female fertility study considered records from very distant periods, that is, from different lactations. As a further challenge for fertility traits compared to milk production is the importance of additional random effects, especially the effect of the service sire on NRR56 and DO. As a novelty in this regard, we developed a customised R script (see Appendix S1), overcoming limitations in modelling further random effects with respective (co)variance structures for longitudinal GWAS.

### 4.2 Candidate Genes and Their Roles in Biological Pathways

We considered as candidate genes those within 100 kb of significant or suggestive SNPs identified in the longitudinal GWAS. The SNP ARS‐BFGL‐NGS‐104247 with significant effects on NRR56 is located in the intron of the *TMEM132C* gene. *TMEM132C* encodes the two transmembrane proteins *ENSBTAP00000055174* and *ESBTAP00000072407*, and is related to the family of genes that are often associated with human genetic disorders, particularly neurological disorders (Sanchez‐Pulido and Ponting 2018). Additionally, this gene has been recently reported in relation to lung function in Tibetans (Zheng et al. 2023), suggesting that *TMEM132C* plays a predominant role with regard to adaptive processes at high altitudes. Furthermore, *TMEM132C* has been associated with the development of the nervous system in rats (Wang et al. 2022), growth traits in pigs (Gong et al. 2019), milk production in Thai multi‐breed cattle (Yodklaew et al. 2017), and milk protein production in Holstein cattle (Liu et al. 2022). In Sarabi cattle, an Iranian taurine breed, the chromosomal segment including *TMEM132C* revealed strong selection signatures (Moradian et al. 2020), suggesting the role of adaptation and natural selection. The study by Souza et al. (2024) indicated associations between *TMEM132C* and body weight in Nellore cattle. Expression of *TMEM132C* in different reproductive organs suggests involvement in reproductive processes by affecting cell–cell interactions within the reproductive tract (Human Protein Atlas 2025). Differential expression of *TMEM132C* has been reported for the mouse nervous system (Wang et al. 2022), where this gene has been shown to influence hormonal signalling pathways that regulate reproductive functions.

*IMPG1* was identified as a potential candidate gene for NRR56. *IMPG1* mutations were causal in 8% of families with adult‐onset vitelliform dystrophy, characterised by moderate visual impairment and drusen‐like lesions in humans (Meunier et al. 2014). Ebrahimi et al. (2023) reported significant downregulation of *IMPG1* in the sperm of infertile men, suggesting a potential role of this protein in sperm functions and male fertility. Although research on *IMPG1* in dairy cattle is limited, its involvement in sperm integrity and expression in reproductive tissues could imply a similar role in bovine fertility. Research in mice suggested a sex‐biased expression in the pituitary gland, indicating a possible regulatory role in reproductive processes, particularly the hypothalamic–pituitary–gonadal axis (Oyola and Handa 2017). Moradian et al. (2020) highlighted *IMPG1* in the context of milk fat percentage, pregnancy rate and milk riboflavin content in Iranian Holstein cattle. Moreover, according to the results from the present study, expressions of *IMPG1* in the fornix of the vagina, uterine cervix and uterine horn, and isthmus of fallopian tube suggest the involvement of *IMPG1* in female fertility processes.

The *DCHS2* gene (identified as a potential candidate gene for CTFS) has been associated with a range of biological processes across different species, particularly in the context of production and reproduction in Simmental cattle (Wang et al. 2025). Hence, our findings align with previous research that highlights its significance in various physiological and reproduction‐related traits. Lodge et al. (2020) reported that mutations in *DCHS2* lead to developmental defects in the pituitary and hypothalamus, resulting in hormone deficiencies and associated health problems in humans. This underscores the fundamental role of *DCHS2* in endocrine regulation, which is crucial for reproductive performance. Similarly, Murugesan et al. (2021) found *DCHS2* to be active during the craniofacial development of buffalo embryos, highlighting its involvement in early developmental processes. Studies in other species support that *DCHS2* influences reproductive and production traits. Gu et al. (2017) and Wu et al. (2022) demonstrated the effects of *DCHS2* variants on reproductive performances in pigs and on weights in humans, respectively. Our results are consistent with Wang et al. (2025), supporting the impact of *DCHS2* on reproductive traits in Simmental cattle. Given its known roles in endocrine functions, craniofacial development and reproductive traits in other species, *DCHS2* could be a potential candidate gene for improving fertility and production efficiency in dairy cattle.

*CSNK1A1* (Casein Kinase 1 Alpha 1), identified as a potential candidate gene for DO, plays a particularly important role in cellular processes such as cell division, differentiation and apoptosis. *CSNK1A1* has been implicated in milk composition traits, including milk fat and protein percentages as well as milk yield, suggesting a potential link to metabolic regulations in high‐producing dairy cows (Ameri et al. 2024). The antagonistic relationship between milk production traits and reproductive performances in cows suggests that *CSNK1A1* may also influence fertility (Ameri et al. 2024). Recent studies have highlighted the role of *CSNK1A1* in uterine gland development, where it regulates epithelial cell apoptosis and the expression of *Foxa2*, a critical factor for uterine function (Zhang et al. 2024). These mechanisms could influence implantation and embryo development, impacting fertility and days open. Additionally, *CSNK1A1* has been shown to regulate the postpartum quiescent state and reproductive cycle in yaks, involving processes such as oocyte maturation, oestrogen and Gonadotropin‐Releasing Hormone (GnRH) signalling (Yang et al. 2024). This suggests that *CSNK1A1* plays a crucial role in reproductive timing and overall fertility, supporting our results from the longitudinal GWAS in dairy cows.

*CSMD1* was identified as a potential candidate gene for DO. Dilower et al. (2023) and Liu et al. (2018) have shown that mice lacking specific protein ligands in granulosa cells fail to develop theca cells, leading to impaired steroidogenesis and infertility. In humans, Jaillard et al. (2016) identified the role of *CSMD1* in ovarian failure in patients under 40 years old, and they proposed this gene as a potential candidate implicated in reproductive functions. Lee et al. (2019) indicated that rare mutations of *CSMD1* cause male and female infertility in humans. Zheng et al. (2023) identified *CSMD1* as a candidate gene related to the body condition score and subcutaneous fat deposition trait in Holstein cattle. Liu et al. (2017) defined *CSMD1* as a candidate gene for fertility traits in Nordic Holstein cows. Gonzalez et al. (2020) found associations between variants of *CSMD1* with conformation traits in dairy cows. Li et al. (2022) highlighted the role of *CSMD1* in fertility mechanisms of Chinese indigenous goat breeds. *CSMD1* was expressed in cumulus cells and oocytes with an expression score larger than 55 (Bastian et al. 2021). The gene expressions in cumulus cells and oocytes further support its role in female fertility, potentially affecting oocyte maturation and quality, which are critical determinants of DO.

Investigations of the biological pathways indicated key biological processes which are directly or indirectly linked to fertility traits, including cell adhesion, hormonal regulation and metabolic functions (Tang et al. 2023). Similar findings were reported in viviparous species, where downregulation of cell adhesion molecules was associated with increased uterine plasticity and preparation for embryo implantation (Whittington et al. 2018). The involvement of *DCHS2* in the Hippo signalling pathway, indicated by an enrichment score of 2.2, further supports its role in reproductive organ development and hormonal regulation. Clark et al. (2022) related Hippo pathway components with important roles in follicle growth and activation as well as with steroidogenesis, by regulating several key biological processes through mechanisms of cell proliferation, migration, differentiation and cell fate determination. In a study addressing the bovine Hippo signalling pathway, Plewes et al. (2019) highlighted the pathway's role in proliferation and estradiol synthesis, which is necessary for maintaining normal follicle development.

*CSNK1A1*, another candidate gene, has been implicated in a broad range of pathways. For example, Yang et al. (2024) described the role of *CSNK1A1* in postpartum quiescence and reproductive cycles of yaks, and upregulation during the seasonal light cycle, which may influence the reactivation of reproductive functions postpartum. Jiang et al. (2018) highlighted that CK1α (the protein encoded by *CSNK1A1*) is active in both the Hedgehog and Wnt pathways, and exhibits high expression in human female reproductive tissues. Taken together, these studies suggest that *CSNK1A1* may enhance reproductive outcomes by regulating processes such as cell division and protein phosphorylation, and the importance of signalling pathways for oocyte development.

Overall, by identifying genes found within genomic regions associated with longitudinal fertility traits, this study enhances our understanding of the underlying biological mechanisms influencing these traits. These traits are influenced by various factors, including gene regulatory mechanisms, hormonal interactions, and metabolic adaptations, highlighting the multifaceted and time‐dependent nature of reproductive performance.

## 5 Conclusions

The longitudinal GWAS enabled a clear separation of SNP effects along the time‐dependent lactation trajectory, that is, for the intercept, for the slope and for the combination of both. The correlation coefficients between SNPs effects for the slope (middle layer) and for the intercept (outer layer) ranged from 0.83 to 0.91 for three fertility traits, reflecting changes in SNP effects over time. These results are supported by declining genetic correlation estimates between the same traits with increasing parity distances. The significant SNPs for the inner layer and for the middle layer, reflecting the dynamic processes of female fertility, explained 0.10%–4.03% of the total genetic variation. These small genetic variances support the polygenic nature of female fertility traits. From the GWAS for the intercept, slope and combined information of the same traits, we identified five potential candidate genes. Gene expression scores of these genes were related to tissues with functions in female fertility processes. Overall, the results from this longitudinal GWAS underscore the importance of specific SNPs, and associated genes, in relation to specific lactations.

## Author Contributions

S.S. performed formal data analysis and wrote the manuscript; T.Y. conceptualised the longitudinal GWAS and supported in programming and statistical analyes, S.K. supervised, developed data anaysis strategies and wrote the manucript.

## Funding

This work was supported by the LOEWE priority program ‘GreenDairy—Integrated Livestock‐Plant‐Agroecosystems’ of the Hessian Ministry of Science and Research, Arts and Culture, grant number LOEWE/2/14/519/03/07.001‐(0007)/80.

## Conflicts of Interest

The authors declare no conflicts of interest.

## Supporting information


**Appendix S1:** age70078‐sup‐0001‐AppendixS1.R.

**Appendix S2:** age70078‐sup‐0002‐AppendixS2.docx.

## Data Availability

Data supporting this study are openly available from the figshare repository at https://figshare.com/s/3b596720d1bbd9282328.
