# Supplementary material for: Longitudinal Genome‐Wide Association Study for Female Fertility Traits in German Holstein Cattle
Source: Anim Genet. 2026 Feb 11;57(1):e70078. doi: 10.1002/age.70078 (PMC12893027; doi:10.1002/age.70078)
Supplement: Supplementary file 2 — Appendix S2: age70078‐sup‐0002‐AppendixS2.docx. [file AGE-57-0-s002.docx]

**Technical note: description of the workflow for the estimation of SNP effects and significances in the longitudinal genome-wide association study**

The estimated (co)variance components from model 1 (see Table 2) defined in step 1 were used as input parameters for the longitudinal GWAS, i.e., **G** =$[\begin{matrix} \boldsymbol{\sigma}_{\boldsymbol{11}} & \boldsymbol{\sigma}_{\boldsymbol{12}} \\ \boldsymbol{\sigma}_{\boldsymbol{21}} & \boldsymbol{\sigma}_{\boldsymbol{22}} \end{matrix}$**]** the variance-covariance matrix for random regression coefficients of additive polygenic effects for intercept and slope; **P** = [$\begin{matrix} \boldsymbol{p}_{\boldsymbol{11}} & \boldsymbol{p}_{\boldsymbol{12}} \\ \boldsymbol{p}_{\boldsymbol{21}} & \boldsymbol{p}_{\boldsymbol{22}} \end{matrix}$**]** the variance-covariance matrix of random regression coefficients for permanent environmental effects for intercept and slope; **S** = $\boldsymbol{\sigma}_{\boldsymbol{s}}^{\boldsymbol{2}}$ variance for the random service sire effect; and **R =** $\boldsymbol{\sigma}_{\boldsymbol{e}}^{\boldsymbol{2}}$ variance for the random residual effect.

1. Calculate ***k*** as the marker-derived genomic relationship matrix **k** = **MM**′ **/ 2∑ pi(1-pi**) with pi = the allele frequency of the *ith* SNP in the centralized variant matrix **M** as defined by VanRaden et al. (2004).
2. Setting up the phenotypic trait (co)variance matrix **W** = **Q**(***k󠄀*** ⊗ **G**)**Q**′ + **Z**(**I** ⊗ **P**)**Z**′ + **I**$\sigma_{s}^{2}$ + **I**$\boldsymbol{\sigma}_{\boldsymbol{e}}^{\boldsymbol{2}}$ with **Q** and **Z** and variances for service sire and residual effects as defined above
3. Eigen decomposition for **W** with **W** = **UDU**′, where **D** is a diagonal matrix containing the eigenvalues, and **U** is the matrix of eigenvectors in the order of the corresponding eigenvalues.
4. Multiplying both sides of **W**= **UDU**′ by **U**' and solving the equation implies the variances for fixed effects and SNP effects: **var([**$\hat{\boldsymbol{b}}$**'** $\hat{\boldsymbol{b}}$**'_SNP_]')** = ([**XX**_SNP_]'**D**^-1^[**XX**_SNP_])^-1^.
5. **y*** = **U**'**y** implies the effect estimates **[**$\hat{\boldsymbol{b}}$**'** $\hat{\boldsymbol{b}}$**'_SNP_]**' = ([**XX**_SNP_]'**D**^-1^[**XX**_SNP_])^-1^[**XX**_SNP_]'**D**^-1^y^*^.

Based on the estimates for the regression coefficients of the additive SNP effects and the corresponding covariance matrix for regression coefficients, the Wald chi-square test for time-independent SNP significance was: $\hat{\boldsymbol{b}}$**'_SNP_ [var(**$\hat{\boldsymbol{b}}$**_SNP_ )]**$\hat{\boldsymbol{b}}$**_SNP_ ~ X^2^(*nf* +1)**, where ***nf*** is the order of basis functions, which equals 1 for the time-varied SNP effect.

Following this approach, the longitudinal GWAS generated three Manhattan plots for the three circular layers. The interpretations of the circular Manhattan plots refer to the effects for the intercept (outer layer Manhattan plot) and slope (middle layer Manhattan). Or, in other words, the outer layer tests whether one SNP has a baseline genetic effect, and the Manhattan plot in the middle layer indicates whether the effect of a SNP changes across lactations. The inner layer illustrates the results of the longitudinal GWAS, highlighting significant SNPs with consistent effects on a trait within all lactations, but where the influence varies across lactations. Consequently, the degrees of freedom (**df**) differed across layers. The outer and middle layer Manhattan plots reflect single tests are based on *df = 1*, whereas the inner layer, as a final result of the longitudinal GWAS, integrated both effects with *df = 2*. In the Manhattan plots for SNP effects, two significant thresholds were considered. As the Bonferroni correction (**𝑃*bonf***) = 0.05 / number of independent test) resulted in a stringent threshold of *𝑝 = 1.2 × 10^-6^* , we opted for a less conservative approach, using a suggestive threshold (**𝑃*sug*** = 0.00005), corresponding to –log_10_(0.00005) = 4.3 (Velayudhan et al., 2023). Finally, we generated Q-Q plots to assess genomic inflation and we applied the significance thresholds to interpret the Manhattan plots for the respective traits and SNP effects.
